# Susceptibility of wild-caught *Lutzomyia longipalpis* (Diptera: Psychodidae) sand flies to insecticide after an extended period of exposure in western São Paulo, Brazil

**DOI:** 10.1186/s13071-019-3364-4

**Published:** 2019-03-14

**Authors:** Mikel A. González, Melissa J. Bell, Scott A. Bernhardt, Reginaldo P. Brazil, Erin Dilger, Orin Courtenay, James G. C. Hamilton

**Affiliations:** 1 Division of Biomedical and Life Sciences, Faculty of Health and Medicine, Lancaster University, Lancashire, LA1 4YG UK; 2 Present Address: Departamento de Sanidad Animal, Instituto Vasco de Investigación y Desarrollo Agrario (NEIKER-Teknalia), Derio, 48160 Biscay Spain; 3 Department of Biology, Utah State University, Logan, Utah 84322 USA; 4 Laboratório de Doenças Parasitárias, Instituto Oswaldo Cruz, Fundaçao Oswaldo Cruz, Av. Brasil, Manguinhos, Rio de Janeiro, RJ 4365 Brazil; 5 School of Life Sciences, University of Warwick, Coventry, CV4 7AL UK

**Keywords:** *Lutzomyia longipalpis*, Insecticide susceptibility, KDT, Sex-aggregation pheromone, Long-exposure, Lambda-cyhalothrin, Brazil

## Abstract

**Background:**

In Brazil, members of the sand fly species complex *Lutzomyia longipalpis* transmit *Leishmania infantum*, a protist parasite that causes visceral leishmaniasis. Male *Lu. longipalpis* produce a sex pheromone that is attractive to both females and males. During a cluster randomised trial, to determine the combined effect of synthetic sex-aggregation pheromone and insecticide on *Le. infantum* transmission *Lu. longipalpis* had been continuously exposed to insecticide for 30 months. The objective of this study was to determine the effect of continuous exposure to the insecticides used in the trial on the susceptibility of *Lu. longipalpis* population.

**Methods:**

During the trial the sand flies had been exposed to either lambda-cyhalothrin [pheromone + residual insecticide spray (PI)], deltamethrin [dog collars (DC)] or no insecticide [control (C)], for 30 months (November 2012 to April 2015). The insecticide treatment regime was kept in place for an additional 12 months (May 2015-April 2016) during this susceptibility study. Sand flies collected from the field were exposed to WHO insecticide-impregnated papers cyhalothrin (0.05%), deltamethrin (0.5%) and control (silicone oil) in a modified WHO insecticide exposure trial to determine their susceptibility.

**Results:**

We collected 788 *Lu. longipalpis* using CDC-light traps in 31 municipalities across the three trial arms. Probit analysis showed that the knockdown times (KDTs) of *Lu. longipalpis* collected from the lambda-cyhalothrin exposed PI-arm [KDT_50_: 31.1 min, confidence interval (CI): 29.6–32.6 and KDT_90_: 44.2 min, CI: 42.1–46.7] were longer than the KDTs from the non-insecticide-treated C-arm (KDT_50_: 26.3 min, CI: 25.1–27.6 and KDT_90_: 38.2, CI: 36.5–40.2) (no-overlapping 95% CIs). KDTs of *Lu. longipalpis* collected from the deltamethrin exposed DC-arm had similar values (KDT_50_: 13.7 min, CI: 10.1–16.2 and KDT_90_: 26.7 min, CI: 21.8–30.6) to those for the C-arm (KDT_50_: 13.5 min; CI: 12.2–14.8 and KDT_90_: 23.2 min, CI: 21.4–25.4) (overlapping CIs). The wild-caught unexposed *Lu. longipalpis* (C-arm), took approximately twice as long to knock down as laboratory-colonised specimens for both insecticides.

**Conclusions:**

Our study reveals slight changes in KDT, in sand flies after prolonged exposure to lambda-cyhalothrin in the presence of pheromone. These changes are not considered to have reached the reference levels indicative of resistance in sand flies suggesting that pheromone and insecticide treatment at the level indicated in this study do not constitute a significant risk of increased insecticide resistance. Prolonged exposure to deltamethrin in dog collars did not result in changes to KDT.

**Electronic supplementary material:**

The online version of this article (10.1186/s13071-019-3364-4) contains supplementary material, which is available to authorized users.

## Background

*Lutzomyia longipalpis* (Diptera: Psychodidae) is the most important vector of *Leishmania infantum*, the protist parasite that causes zoonotic visceral leishmaniasis (VL) in the Americas. In São Paulo (SP) State, Brazil, VL is an emerging disease, and *Lu. longipalpis* was first reported in Araçatuba, a city in the west of the state, in 1997 [1]. Between 1999 and 2013, up to 2324 human cases and 200 deaths were recorded in SP, which corresponds to an incidence rate of 2.8 cases and mortality of 0.2 deaths per 100,000 inhabitants per year equivalent to an 8.6% case fatality rate [2]. Since then, VL has become endemic in the Araçatuba region, which along with Bauru is considered to be the main focus of the disease in SP [2]. Over the past few years *Lu. longipalpis* has expanded its distribution across the state with a consequent increase in the number of municipalities reporting canine and human transmission [3–5].

Current strategies for the prevention and control of VL recommended by the Brazilian Ministry of Health (BMH) include both canine and vector control strategies [5]. Vector control, by indoor residual spraying (IRS) of pyrethroids (e.g. deltamethrin, lambda-cyhalothrin and cypermethrin) [6] in human dwellings and animal shelters, is still considered by many health authorities to be the most effective method for rapidly reducing vector populations [7]. Current recommendations in Brazil are for reactive IRS of households within a 200 m radius of a reported human VL case [5]. IRS is assumed to help reduce VL burden; however, there is little direct empirical evidence for this [8], as it depends on reduced biting behaviour of the vector [9] and compliant human behavioural response to insecticide-based protective measures [10].

*Lutzomyia longipalpis* has a strong association with chickens; both as an important food source and because poultry shelters are used as resting areas and aggregation sites [11]. This makes poultry shelters a target for sand fly control. Although the precise role of chickens in the epidemiology of leishmaniasis is unclear, the presence of chickens and other animals (birds and mammals) is variably cited as a risk factor for human infection [12].

*Lutzomyia longipalpis* uses a sex-aggregation pheromone, released by males, to create aggregations of males and blood-meal seeking females on or near chickens or other animals [11]. Recently, it was shown that the synthetic sex-aggregation pheromone of *Lu. longipalpis* in western São Paulo State, [(±)-9-methylgermacrene-B], attracts both sexes of *Lu. longipalpis* to insecticide-treated chicken sheds resulting in increased sand fly mortality [13]. When used without synthetic pheromone, the insecticide kills the males that arrive first at insecticide-treated sites and thus disrupts their pheromone release. As any further recruitment of either males or females to the insecticide-treated site is interrupted the development of aggregations in alternative non-insecticide-treated locations, e.g. on potentially unprotected hosts may occur [11, 13]. Synthetic sex pheromone can overcome this problem by maintaining the recruitment and loyalty of both male and female sand flies to insecticide-treated sites over a prolonged period of time thereby ensuring contact between the sand flies and insecticide without the risk of aggregations forming near unprotected hosts [13, 14].

Insecticide-impregnated collars fitted to dogs (the reservoir of *Le. infantum*) is an alternative vector intervention which has been shown to reduce prevalence and incidence of infection in canines [15], reduce infection incidence in children [16], and reduce sand fly densities [17]. Dog collars are easy to apply and generally well-accepted by dog owners [18] and under certain conditions are predicted to be more efficient than either canine vaccination or euthanasia to reduce transmission [19]. In Brazil, although insecticide-impregnated dog collars are expensive they are being purchased with increased frequency by some municipalities although the required coverage to reduce transmission in the uncollared dog population is unknown.

A consequence of increasing *Lu. longipalpis* exposure to insecticide through their programmatic deployment, either along with synthetic sex-aggregation pheromone or in the widespread use of insecticide-impregnated dog collars, is the risk of inducing insecticide resistance.

Although several studies have reported on the susceptibility of laboratory-reared sand fly colonies to insecticides [20, 21], there are few that focus on susceptibility of wild populations to insecticides and most of them are on the Old World *Phlebotomus* species [22–26]. These field studies often lack information on previous insecticide exposure, report non-standardized methodologies or unknown or varied insecticide concentrations and times of exposure.

Although the development of resistance of *Lu. longipalpis* to agricultural and mosquito control insecticides has been reported in some areas of Brazil and Venezuela [27, 28], the current practise of reactive IRS in response to human VL cases is geographically discontinuous, temporally sporadic, variably sustained [4, 5] and because of the disruptive effect on *Lu. longipalpis* aggregation behaviour is unlikely to lead to insecticide resistance.

In this study, *Lu. longipalpis* sand flies were exposed to two field-based experimental vector control interventions for 30–39 months; lambda-cyhalothrin + synthetic sex-aggregation pheromone applied to householder’s chicken sheds or deltamethrin impregnated dog collars applied to householder’s dogs. Our aim was to determine if prolonged exposure to insecticide altered the susceptibility of *Lu. longipalpis* in the Araçatuba study area to either lambda-cyhalothrin or deltamethrin.

## Methods

### Study area

The study was carried out in the mesoregion of Araçatuba, (21°12′32″S, 50°25′58″W, altitude 390 m above sea level) approximately 530 km west of the city of São Paulo, in 31 semi-urban municipalities, towns and villages located in an area of 16,768 km^2^ with a population of approximately 696,000 inhabitants.

Based on the Köppen climate classification, this region is classified as Aw type, with dry winters and hot rainy summers [29]. The region is endemic for both canine and human VL [30, 31].

The houses in the study were primarily constructed of brick with tiled or corrugated roof and were located in non-paved urban and peri-urban settings. Households typically contained domestic animals (e.g. predominantly chickens, dogs, cats and occasionally pigs, sheep or horses), small shrubs and bushes, vegetable gardens and fruit trees.

### Study design

The study design followed exactly the design of a cluster-randomised trial (CRT) against *Le. infantum* canine infection incidence. In that trial, selected houses within towns of the Araçatuba region and suburbs of Araçatuba city (clusters) had been randomised to receive one of three treatments: (i) pheromone + insecticide (PI); (ii) insecticide-impregnated dog collars (DC); or (iii) sham control (C) over a period of 30 months. At the end of the trial, the insecticide interventions were continued for a further 12 months and sand flies were sampled from each of the treatment arms for this insecticide susceptibility study. In total, the insecticide interventions were in place for 42 months, from the beginning of November 2012 to the end of April 2016.

### Insecticide treatments

During the period of this study, the insecticide interventions that had been used in the CRT were continued. In the PI-arm, chicken shelters and roosting sites were sprayed with microencapsulated lambda-cyhalothrin (20 mg a.i. m^-2^; Demand CS; BASF, Cheshire, UK) following the guidelines and recommendations of the BMH every three months. Lambda-cyhalothrin is a long-lasting insecticide recommended by the BMH for phlebotomine sand fly control [5]. It has a 100% lethal efficacy within the first 24 h and an acceptable lethality for at least six months after application although some reduction in effectiveness can be observed during this time depending on the type of surface on which it is sprayed [32–34]. In addition, a pheromone dispenser (lure) loaded with 10 mg of the synthetic sex pheromone, (±)-9-methylgermacrene-B, was attached near to the chicken shelters in order to attract sand flies to the insecticide-treated surfaces. The lures, which are attractive to both sexes for up to three months [35], were replaced every three months. The insecticide spray treatment was reapplied every six months.

In the DC-arm, dogs were fitted with a collar containing 1.0 g of deltamethrin (Scalibor Dog Collar, Intervet Productions S.A., France). Dog collars have been shown to be effective in reducing the sand fly infestation levels near dogs and the number of sand flies biting dogs on which they are fitted [17]. Collars were replaced every six months according to the manufacturer’s instructions.

In the C-arm, chicken shelters were sprayed with water instead of insecticide and a mock lure without pheromone was fitted. A plastic collar without insecticide was also fitted on the dogs. All of placebo treatments were carried out with the same frequency as for the PI and DC-arms.

In each study treatment cluster, 5–8 households which reported no previous significant exposure to insecticide, were randomly recruited with informed consent, giving a total of 226 sampled households from the 547 treated households available (Additional file 1: Table S1). As a consequence of the original CRT recruitment criteria, all households in this study had one or more dog(s) and chicken(s) resident.

The study began in May 2015, 30 months after the start of the CRT insecticide applications, and ended in April 2016. Sand fly trapping was conducted in the four insecticide/control treatment rounds (13–16) and each trapping period lasted for 13 days (95% CI: 8–17). Traps were set for a single night per dwelling. In total, 388 traps were set on 52 non-consecutive trapping nights in 31 treatment clusters, equally distributed between the three treatment arms (Additional file 1: Table S2).

### Sand fly collection

*Lutzomyia longipalpis* sand flies were trapped with Centers for Disease Control (CDC) miniature light traps equipped with standard incandescent light bulbs. The CDC traps were hung between 1 and 4 m from the insecticide-treated areas (dependant on availability of structures to suspend the traps), i.e. in the chicken shelter for the PI-arm, in or near the collared dog kennel/bed for the DC-arm and at different positions near the chicken shed for the C-arm. Traps were suspended at dusk and retrieved the next morning (08:00 h) in the same way for all three arms. Nylon mesh bags containing insects were immediately transported from the field locations to the laboratory.

The previous CRT had established that the *Lu. longipalpis* sand flies in the Araçatuba mesoregion produce the 9-methylgermacrene-B sex/aggregation pheromone. Periodic sampling during this study confirmed that the *Lu. longipalpis* were of the same pheromone type.

### Laboratory colony

A laboratory colony of *Lu. longipalpis* with no recent exposure history (i.e. in the previous 8–9 years) to insecticides was used as a laboratory reference strain to test susceptibility to lambda-cyhalothrin and deltamethrin in World Health Organisation (WHO) insecticide tests. The colony which was maintained as previously described [36], was established from males and females originally collected in Campo Grande (Mato Grosso do Sul, Brazil) in 2007. The colony was in the 73rd-82nd generation when the study was carried out. Males of this population produce the same sex pheromone, (*S*)-9-methylgermacrene-B, as the wild specimens from the study area [37].

### Insecticide exposure tests

Field-collected *Lu. longipalpis* specimens were released into a cage 30 min prior to being tested to acclimatize. Only specimens that appeared undamaged and in good physical condition (i.e. capable of walking, climbing and flying) were used for the insecticide exposure tests.

Instead of WHO tubes (174 cm^3^, 11 cm long, 4.5 cm in diameter) which were not available for this study, we used similarly sized but readily available plastic tubes (147 cm^3^, 7.5 cm long, 5.0 cm in diameter). This allowed us to measure the relative changes to insecticide susceptibility by comparing knockdown times (KDTs) between insects collected from the three arms and the laboratory colony [38], but not standard reference exposure dosages.

The sand flies were exposed to WHO test kit standard insecticide-impregnated papers obtained from the Vector Control Research Unit (Universiti Sains Malaysia, Malaysia): lambda-cyhalothrin (0.05%), deltamethrin (0.5%) and control (silicone oil). These had been reduced to roughly half their original size (12 × 5 cm) and were used to line the inner surface of the tubes. Sand flies were aspirated from the nylon mesh holding cages and gently introduced into the tubes through a small hole in the nylon mesh screen that covered the open end. A small piece of cotton wool was inserted in the hole to prevent the flies from escaping. The experimental tubes were placed horizontally on a lab bench in a room at 25 ± 2 °C and 80 ± 10% relative humidity in accordance with WHO protocols [39]. Insecticide impregnated papers were used no more than three consecutive times within a maximum of five days after unpacking. In this study, tubes with insecticide papers inside were wrapped in aluminium foil and stored in a shaded area until the next bioassay to prevent degradation or loss of insecticide.

The numbers of sand fly specimens collected in the Araçatuba region were generally low and variable over time (Table 1). Therefore, as the susceptibility tests depended on the availability of sand flies, these were carried out on all available collected specimens regardless of their physiological status (fed/unfed), age and sex. As a result almost three times more males were tested than females, this ratio was similar to the usual male to female ratios captured by CDC light traps [13]. This proportion was kept constant for each insecticide test round. KDTs were noted at 5 min intervals over 60 min. Specimens were classified as “knocked down” according to the criteria defined by the WHO [39]; “dead, immobile, unable to stand or fly in a coordinated way”.

**Table 1:** Total numbers of *Lu. longipalpis* collected in each of the three treatment arms during the four rounds of the insecticide susceptibility experiments in Araçatuba and surrounding municipalities (SP, Brazil)

Year	2015		2016		Total
Date of collection	20/7-10/8	15/10-5/11	12/1-27/2	11/4-3/5	
Round	13	14	15	16	
PI-arm	20	20	40	185	265
DC-arm	33	29	92	120	274
C-arm	59	39	80	71	249
Total	112	88	212	376	788

*Abbreviations*: PI-arm, pheromone insecticide arm; DC-arm, dog-collar arm; C-arm, control arm

Dates indicate start and end periods of sand fly trapping within each round

After one hour of exposure, live sand flies (including dying individuals) were transferred to new containers and held at the same temperature and humidity environment as before for the susceptibility trials, for a period of 24 h. A cotton ball saturated with 40% sugar-water was placed on the top of each container to provide a food source. The total number of sand flies from each arm collected and exposed to the insecticide paper is presented in Table 1.

Colonised sand flies were treated in the same manner as the wild-caught sand flies although the susceptibility assays were performed in an insectary, maintained at 27 ± 2 °C and 80 ± 15% rh, in UK. For the laboratory colony experiment, six groups of 20 unfed specimens (5–7 days-old) were tested for each insecticide concentration. The gender ratio used in these experiments was the same as the wild-caught sand flies.

Finally, two groups of 25 *Lu. longipalpis* one collected from the C-arm and the other from the laboratory colony were tested in tubes with silicone control papers only to exclude any possible toxic effect of silicone. Sand flies from the control arm (*n* = 25) were tested depending on their availability from the field collections while laboratory colony specimens (*n* = 25) were tested in small groups for the six replicates.

### Sand fly identification

At the end of each test, sand fly species identity was confirmed with appropriate identification keys [40] by mounting the male genitalia or female spermatheca on glass slides with Canada balsam and examining under a microscope (Quimis Ltda., Sao Paulo) at 40× magnification.

### Statistical analysis

Data from the KDT-response tests were analysed by probit analysis (log-probit) to determine the time to knockdown 50% and 90% of the population (KDT_50_ and KDT_90_) and their confidence intervals (CI) (v15.0, SPSS Inc.) according to standard WHO testing protocols [39]. Regression parameters and the chi-square test were calculated using the output file of probit analysis for each insecticide (Additional file 1: Table S2). These data generated a KDT-response analysis from which the time necessary to knockdown 50 or 90% (KDT_50_ and KDT_90_, respectively) of the field-collected populations, and were compared to each other and to the laboratory colony sand flies. 95% CI were used to detect overlapping between groups. Mortality was observed 24 h after the 60 min insecticide exposure. As the control group mortality was < 5% we did not correct for control mortality rates [41].

## Results

Morphological examination of the genitalia confirmed that all the specimens were either *Lu. longipalpis* (98.1%) or *Brumptomyia* spp. (1.9%). The *Brumptomyia* spp. were excluded from further analysis.

In total, 788 wild-caught *Lu. longipalpis* from across the three arms of the experiment and 240 from the laboratory colony were tested for their susceptibility to insecticides (Table 1).

All wild specimens were knocked down within 60 min of exposure to lambda-cyhalothrin (0.05%) or deltamethrin (0.5%), although 6.6% of the laboratory colony specimens survived 60 min exposure to lambda-cyhalothrin, these died during the subsequent 24 h holding period.

**Fig. 1:**
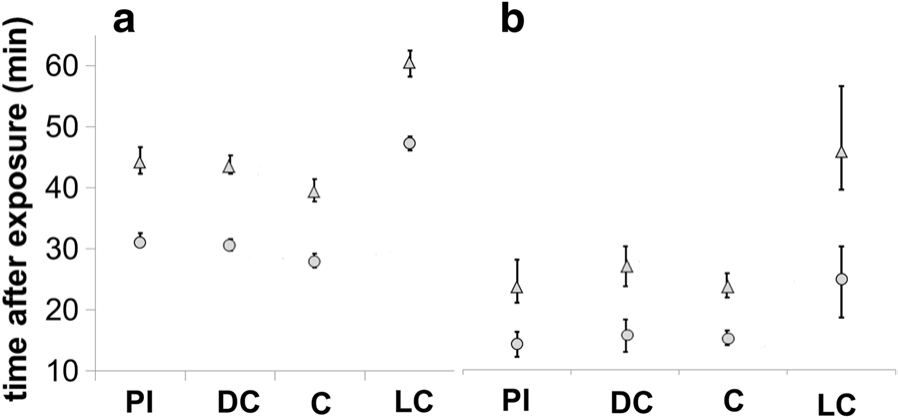
Knockdown times of *Lu. longipalpis* sand flies exposed to 0.05% lambda-cyhalothrin (**a**) and 0.5% deltamethrin (**b**) for a period of 60 min. KDT_50_ and KDT_90_ are represented with circles and triangles, respectively. The bars above and below the symbols denote the 95% confidence intervals. Data shown are the pooled numbers of sand flies collected in rounds 13–16. *Abbreviations*: PI, pheromone-insecticide arm; DC, dog-collar arm; C, control arm; LC, laboratory colony

**Fig. 2:**
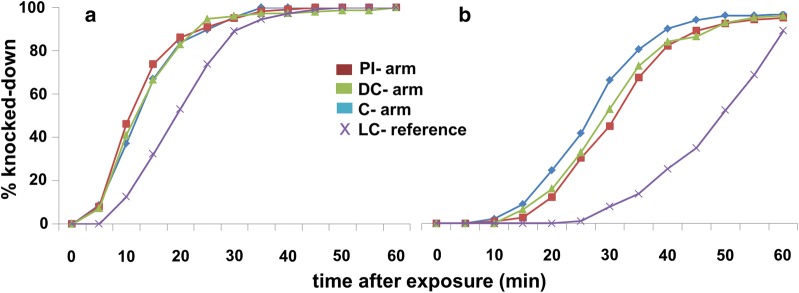
Knockdown times over 60 min in response to exposure to deltamethrin (**a**) and lambda-cyhalothrin (**b**) of *Lu. longipalpis* from the three different treatments. *Abbreviations*: PI, pheromone-insecticide arm; DC, dog-collar arm; C, control arm; LC, laboratory colony

Of the 265 *Lu. longipalpis* collected from the PI-arm, 134 were tested for lambda-cyhalothrin and 131 for deltamethrin susceptibility, of the 274 collected from the DC-arm, 132 were tested for lambda-cyhalothrin and 142 for deltamethrin susceptibility and of the 249 collected from the C-arm, 115 were tested for lambda-cyhalothrin and 134 for deltamethrin susceptibility. The numbers of *Lu. longipalpis* tested from the four sampling rounds (13–16) were 112, 88, 212 and 376, respectively (Table 1). KDT_50_ and KDT_90_ for all *Lu. longipalpis* sand flies exposed to lambda-cyhalothrin (0.05%) and deltamethrin (0.5%) for a period of 60 min is given in Fig. 1. Representative knockdown curves of the sand flies collected in these four rounds are presented in Fig. 2. The total KDT_100_ in the PI-arm was 60 min (lambda-cyhalothrin) and 30 min (deltamethrin), in the DC-arm was 45 min (lambda-cyhalothrin) and 35 min (deltamethrin) and in the C-arm was 55 min (lambda-cyhalothrin) and 40 min (deltamethrin).

*Lutzomyia longipalpis*, previously exposed to lambda-cyhalothrin (PI-arm) had slightly longer KDT_50_ and KDT_90_ times than those collected from the DC and C arms. *Lutzomyia longipalpis* from the PI-arm had KDT_50_ values of 31.1 min (CI: 29.6–32.6) compared to 29.9 min (CI: 28.9–30.8) for DC-arm and 26.3 min (CI: 25.1–27.6) for the C-arm. These results indicate a difference in response as the 95% CI do not overlap one another. *Lutzomyia longipalpis* collected in the PI-arm also had higher KDT_90_ values: 44.2 min (CI: 42.1–46.7) compared to 43.1 min (CI: 41.7–44.6) for the DC-arm and 38.2 min (CI: 36.5–40.2) for the C-arm. Similarly, the 95% CI of the PI-arm does not overlap with C-arm (Fig. 1).

By comparison, there was no difference in the susceptibility of sand flies exposed to deltamethrin compared to those that were not exposed. *Lutzomyia longipalpis* collected from the DC-arm (exposed to deltamethrin), had similar KDT_50_ values (13.7 min, CI: 10.1–16.2) to those collected in the PI-arm (12.8 min, CI: 10.4–15.0) and the C-arm (13.5 min, CI: 12.2–14.8). The 95% CI values of these three groups overlapped. The KDT_90_ values for the DC-arm were slightly higher (26.7 min, CI: 21.8–30.6) than those for the PI-arm (23.3 min, CI: 20.5–27.3) and the C-arm (23.2 min, 21.4–25.4) again the CIs overlapped indicating negligible difference between the knockdown of sand flies despite some prior environmental exposure (Fig. 1).

Sand flies from the laboratory colony were more tolerant for both insecticides compared to the field-collected sand flies. Overall, the laboratory reared sand flies took almost 2 times longer (both KDT_50_ and KDT_90_) to knock down than wild-type (Figs. 1, 2).

## Discussion

Our study showed a slight difference in the KDTs of *Lu. longipalpis* that had been exposed over 30–42 months to lambda-cyhalothrin in sex-aggregation pheromone + residual insecticide-treated chicken roosting sites compared to those that were unexposed in the mesoregion of Araçatuba. By comparison of these KDT changes with those reported in other Old and New World sand fly species on exposure to different insecticides, we consider the changes that we observed to be small and unlikely to represent meaningful alterations in susceptibility. No perceptible differences in KDTs were observed between those sand flies exposed to deltamethrin-impregnated dog collars and those that had not been exposed.

Data on the status of resistance and susceptibility of the Old World sand fly genus *Phlebotomus*, and in particular, *P. papatasi* and *P. argentipes* [24] is extensive; however, there is a lack of similar studies on the New World genera. Within the genus *Lutzomyia*, and with the exception of some studies on *Lu. youngi* [42] and *Lu. evansi* [43], the focus of attention has been directed towards *Lu. longipalpis,* with most, but not all [27, 44, 45] of these studies on *Lu. longipalpis* from Brazil and in particular from Minas Gerais State [20, 21, 28, 46].

*Lutzomyia longipalpis* populations repeatedly exposed to pyrethroids in VL and dengue control programmes have been shown to be less susceptible than unexposed populations in modified WHO tube assays [28]. The same populations showed no changes in susceptibility to organophosphates [28]. A similar, decrease in susceptibility to three commonly used agricultural insecticides was found in *Lu. longipalpis* from Venezuela [27].

In our study, we used the WHO Pesticide Evaluation Scheme discriminating dosage for lambda-cyhalothrin and were therefore able to compare the KDTs that we obtained directly with those obtained in other studies. The differences in KDTs between exposed and unexposed populations of sand flies seen in our study were much less than those seen in other studies. In our CRT, the KDT_50_ and KDT_90_ were each 1.2 times higher respectively in the exposed (PI-arm) than unexposed (C-arm) populations. By comparison, *P. sergenti* collected in one area of Morocco had KDT_50_ and KDT_90_ values, when exposed to lambda-cyhalothrin papers (0.05%), that were 2–3 times lower than *P. sergenti* collected in another area [22].

Another study in western Turkey showed that wild-collected *Phlebotomus* and *Sergentomyia* sand flies previously exposed to deltamethrin and permethrin in mosquito control programmes were approximately 2 times less susceptible (KDT_50_ but not KDT_95_ values) than those that were previously unexposed [23]. Again the difference in KDT between these populations was greater than the difference seen in our study, where KDT_50_ and KDT_90_ of *Lu. longipalpis* exposed to deltamethrin in the DC-arm were only 1 and 1.2 times respectively higher, than in the C-arm. Although only a small fraction of dogs in the study area were treated with dog-collars, these results suggest that the mass use of dog collars in the prevention and control of canine leishmaniasis might not contribute significantly to development of insecticide-resistance.

Thus, the differences in susceptibility observed between populations exposed to either lambda-cyhalothrin or deltamethrin in our study seem lower than those observed in other works, suggesting that the differences that we observed are of minor importance. Also, in both the Turkish and Moroccan studies the confidence intervals between both localities and countries were very variable which was also observed here. This might reflect intraspecific heterogeneity of insecticide susceptibility tests and therefore the difficulty of interpreting the results derived from wild populations.

Another interesting finding was the differences in KDT between the laboratory colony reference sand flies and the unexposed wild-caught control arm specimens. Laboratory-colonised sand flies have consistently been found to be less susceptible to insecticides than unexposed wild-caught specimens. The phenomenon was described previously [27, 47] and it may be that vigour tolerance is conferred by the improved nutritional state and comparatively larger body size of colony reared sand flies [48]. Although it is also possible that the trapping, handling and transport from the field could have contributed to an weakened physical condition and thus, reduced their response in the assays. Our results suggest that unexposed populations of wild-caught sand flies should be used as controls for wild-caught exposed populations and not laboratory strains.

Regional-scale field studies involving the systematic application of insecticide over a long period of time are rare because they are difficult to implement and are subject to methodological and other variations which ultimately limit comparisons, e.g. (i) the discriminating concentrations and baseline susceptibility times to different insecticides for *Lu. longipalpis* colonies have not been fully established [21, 24, 46]. Although it is reasonable to use those from similar genera, they cannot be extrapolated from other insect groups such as *Anopheles* mosquitoes [26, 39, 49]. We followed the WHO recommendations for lambda-cyhalothrin (0.05%) and used a higher concentration of deltamethrin (0.5%) as suggested by other authors for different sand fly species [43, 50, 51]; (ii) the methods used in different published studies are not identical (e.g. different insecticide concentration, types of WHO or CDC tests, and time of exposure); (iii) the low numbers of sand flies in the Araçatuba region was overcome by trapping over multiple nights but this may have exposed them to differing levels of stress; (iv) the effect of the chosen sampling technique; (v) unknown characteristics of the sand flies collected such as age, sex and physiological status could have influenced the results of the susceptibility tests [24, 25, 39]. These problems could be partially overcome by either using adults derived from larval collections (although this would be virtually impossible with sand flies) or by rearing the sand flies in the laboratory from wild-caught female sand flies and using the F1 progeny [39]. However, sand fly colonies are difficult to establish and maintain, and this probably accounts for the lack of studies to monitor insecticide resistance. Finally, (vi) the study used exposure containers which differed from the standard WHO kits. Although the size and shape of the exposure containers used in the present study were very similar to the WHO test kit, variation is not uncommon depending on local circumstances [28] and in our case the impregnated test papers were supplied by the WHO and therefore, KDT should be very similar for sand flies despite differences in volume of the container [21].

## Conclusions

This study suggested that there was no substantial change in the susceptibility of the *Lu. longipalpis* population after long-term exposure to residual insecticide. This suggests that using the synthetic sex-aggregation pheromone to attract *Lu. longipalpis* to insecticide-treated sites in a lure-and-kill vector control strategy would not substantially increase the risk of resistance development. In our study, only approximately 20% of households were treated with pheromone and insecticide and, if a greater proportion of households were treated, the outcome might differ. In any case, as *Lu. longipalpis* possesses the mechanisms for development of resistance as previously noted, therefore the early adoption of possible loss of tolerance strategies for pheromone-based lure-and-kill programmes might be considered. The dog-collar intervention, provided no evidence for ongoing loss of susceptibility at this time. Studies on molecular mechanisms of insecticide resistance such as identification of molecular markers and biochemical assays are also needed. Further research, in this area should be based on laboratory progeny obtained from field-caught sand flies, to ensure that results are based on individuals with similar physiological status and in well-controlled conditions. This study highlights the importance of evaluating the impact of repeated exposure of *Lu. longipalpis* adults to insecticide to inform local and national health authorities on the long-term impact of using the synthetic sex-aggregation pheromone as a part of a new potential control measure for sand flies. Finally, it is also important to keep in mind the rising incidence of VL during the last few decades together with the recent intensive spraying programmes against *Aedes aegypti* in most areas of Brazil that are causing an increasing selective pressure on pyrethroids, leading to greater exposure to a wide array of chemicals for all vectors, including *Lu. longipalpi*s.

## Additional file

**Additional file 1: Table S1.** The number of houses sampled with CDC-light traps from July 2015 to April 2016 in each of the four rounds (13–16) of the three intervention trial arms in all the municipalities (towns and villages and Aracatuba neighbourhoods) of Araçatuba and surroundings (SP, Brazil). **Table S2.** Parameters of the probit regression lines determined from KDT experiments with pyrethroids for *Lu. longipalpis* sand flies from the mesoregion of Araçatuba (SP, Brazil).

## Abbreviations

BMH: Brazilian Ministry of Health
CDC: Centers for Disease Control
CI: Confidence interval
CRT: Cluster-randomised trial
IRS: Indoor residual spraying
KDTs: Knock-down times
SP: São Paulo
VL: Visceral leishmaniasis
WHO: World Health Organization
