# Supplementary material for: Susceptibility of wild-caught Lutzomyia longipalpis (Diptera: Psychodidae) sand flies to insecticide after an extended period of exposure in western São Paulo, Brazil
Source: Parasit Vectors. 2019 Mar 14;12:110. doi: 10.1186/s13071-019-3364-4 (PMC6419423; doi:10.1186/s13071-019-3364-4)
Supplement: Supplementary file 1 — Additional file 1: Table S1. The number of houses sampled with CDC-light traps from July 2015 to April 2016 in each of the four rounds (13–16) of the three intervention trial arms in all the municipalities (towns and villages and Aracatuba neighbourhoods) of Araçatuba and surroundings (SP, Brazil). Table S2. Parameters of the probit regression lines determined from KDT experiments with pyrethroids for Lu. longipalpis sand flies from the mesoregion of Araçatuba (SP, Brazil). [file 13071_2019_3364_MOESM1_ESM.docx]

**Additional file 1**

| **R** | **PI** | **N** | **coordinates** | **DC** | **N** | **coordinates** | **C** | **N** | **coordinates** | **T** |
| --- | --- | --- | --- | --- | --- | --- | --- | --- | --- | --- |
| **13** | **Araçatuba (1)** | 8 | 21 °12 ' 32 " S, 50 ° 25 ' 58 " W | **Bento de Abreu** | 3 | 21 ° 16 ´ 14 ´´, 50 ° 48 ´ 43 ´´ | **Brauna** | 7 | 21 ° 29 ´ 57 ´´, 50 ° 18 ´ 56 ´´ | **56** |
|  | **Brejo Alegre** | 6 | 21 ° 09 ´ 59 ´´, 50 ° 11 ´ 07 ´´ | **Araçatuba (3)** | 6 | 21 ° 12 ´ 32 ´´, 50 ° 25 ´ 58 ´´ | **Araçatuba (4)** | 7 | 21 ° 12 ´ 32 ´´, 50 ° 25 ´ 58 ´´ |  |
|  | **Bilac** | 6 | 21 ° 28 ´ 10 ´´, 50 ° 28 ´ 14 ´´ | **Birigui** | 8 | 21 ° 17 ´ 19 ´´, 50 ° 20 ´ 24 ´´ | **Valparaíso** | 5 | 21 ° 13 ´ 40 ´´, 50 ° 52 ´ 06 ´´ |  |
|  |  |  |  |  |  |  |  |  |  |  |
| **14** | **Araçatuba (2)** | 7 | 21 ° 12 ´ 32 ´´, 50 ° 25 ´ 58 ´´ | **Rubiácea** | 7 | 21 ° 18 ´ 02 ´´, 50 ° 43 ´ 36 ´´ | **Brauna** | 7 | 21 ° 29 ´ 57 ´´, 50 ° 18 ´ 56 ´´ | **60** |
|  | **Guararapes** | 6 | 21 ° 15 ´ 39 ´´, 50 ° 38 ´ 34 ´´ | **Birigui** | 6 | 21 ° 17 ´ 19 ´´, 50 ° 20 ´ 24 ´´ | **Penápolis** | 6 | 21 ° 25 ´ 11 ´´, 50 ° 04 ´ 39 ´´ |  |
|  | **Sâo José** | 7 | 21 ° 12 ´ 32 ´´, 50 ° 26 ´ 51 ´´ | **Glicério** | 7 | 21 ° 22 ´ 34 ´´, 50 ° 12 ´ 21 ´´ | **Araçatuba (5)** | 7 | 21 ° 12 ´ 32 ´´, 50 ° 25 ´ 58 ´´ |  |
|  |  |  |  |  |  |  |  |  |  |  |
| **15** | **Guararapes** | 7 | 21 ° 15 ´ 39 ´´, 50 ° 38 ´ 34 ´´ | **Salmourão** | 6 | 21 ° 37 ´ 27 ´´, 50 ° 51 ´ 38 ´´ | **Rinópolis** | 7 | 21 ° 43 ´ 33 ´´, 50 ° 43 ´ 20 ´´ | **56** |
|  | **Auriflama** | 6 | 20 ° 41 ´ 08 ´´, 50 ° 33 ´ 17 ´´ | **Barbosa** | 6 | 21 ° 16 ´ 00 ´´, 49 ° 56 ´ 57´´ | **Santópolis do Aguapeí** | 6 | 20 ° 38 ´ 15 ´´, 50 ° 30 ´ 01´´ |  |
|  | **Mirandopolis** | 6 | 21 ° 08 ´ 01 ´´, 51 ° 06 ´ 06 ´´ | **Guaraçaí** | 6 | 21 ° 01 ´ 42 ´´, 51 ° 12 ´ 24 ´´ | **Andradina** | 6 | 20 ° 53 ´ 46 ´´, 51 ° 22 ´ 46 ´´ |  |
|  |  |  |  |  |  |  |  |  |  |  |
| **16** | **Murutinga do Sul** | 6 | 20 ° 08 ´ 36 ´´, 51 ° 16 ´ 39 ´´ | **Avahandava** | 6 | 21 ° 27 ´ 39 ´´, 49 ° 56 ´ 59 ´´ | **Sud Mennucci** | 6 | 20 ° 41 ´ 27 ´´, 50 ° 55 ´ 26 ´´ | **54** |
|  | **Alto Alegre** | 6 | 21 ° 34 ´ 50 ´´, 50 ° 09 ´ 49 ´´ | **Lavinia** | 6 | 21 ° 10 ´ 06 ´´, 51 ° 02 ´ 23 ´´ | **Andradina** | 6 | 20 ° 53 ´ 46 ´´, 51 ° 22 ´ 46 ´´ |  |
|  | **Castilho** | 6 | 20 ° 52 ´ 20 ´´, 51 ° 29 ´ 15 ´´ | **Barbosa** | 6 | 21 ° 16 ´ 00 ´´, 49 ° 56 ´ 57´´ | **Nova Lourdes** | 6 | 20 ° 51 ´ 22 ´´, 50 ° 15 ´ 42 ´´ |  |

**Additional file 1: Table S1.** The number of houses sampled with CDC-light traps from July 2015 to April 2016 in each of the four rounds (13-16) of the three intervention trial arms in all the municipalities (towns and villages and Aracatuba neighbourhoods) of Araçatuba and surroundings (SP, Brazil).

R = rounds; PI = pheromone-insecticide arm, DC = dog-collar arm, C = control arm; N = number of houses sampled; T = total; the numbers in brackets after Araçatuba indicate the different neighbourhoods within Araçatuba city where sampling was carried out: (1) Alvorada, (2) Morada dos Nobres, (3) various neighbourhoods, (4) Umuarama, Etemp (3).

**Additional file 1: Table S2.** Parameters of the probit regression lines determined from KDT experiments with pyrethroids for *Lu. longipalpis* sand flies from the mesoregion of Araçatuba (SP, Brazil).

| **CRT arm ^a^** | **Insecticide test** | **a** | **b ± SE** | **χ2 (df)** | ***P* -value** | **y = a + bx** |
| --- | --- | --- | --- | --- | --- | --- |
| **PI** | L (0.05%) | -2.851 | 0.108 ± 0.005 | 20.7 (11) | 0.036 | -2.851 + 0.108 x |
|  | D (0.5%) | -1.571 | 0.122 ± 0.006 | 61.4 (11) | 0.000 | -1.57.1 + 0.122 x |
|  |  |  |  |  |  |  |
| **DC** | L (0.05%) | -2.909 | 0.097 ± 0.004 | 16.7 (11) | 0.116 | -2.909 + 0.097 x |
|  | D (0.5%) | -1.589 | 0.117 ± 0.005 | 146.8 (11) | 0.000 | -1.589 + 0.117 x |
|  |  |  |  |  |  |  |
| **C** | L (0.05%) | -3.056 | 0.098 ± 0.007 | 22.1 (11) | 0.023 | -3.056 + 0.098 x |
|  | D (0.5%) | -1.808 | 0.133 ± 0.004 | 27.3 (11) | 0.004 | -1.808 + 0.133 x |
|  |  |  |  |  |  |  |
| **LC** | L (0.05%) | -4.382 | 0.092 ± 0.005 | 7.7 (11) | 0.740 | -4.382 + 0.092 x |
|  | D (0.5%) | -1.477 | 0.060 ± 0.003 | 132.1 (1) | 0.000 | -1.477 + 0.060 x |

^a^ Pheromone insecticide arm (PI), dog-collar arm (DC), control arm (C) of the CRT and *Lu. longipalpis* laboratory colony (LC). L = lambda-cyhalothrin and D = deltamethrin, a = intercept, b ± SE = slope and standard error, χ^2^ (df) = heterogeneity and degrees of freedom.
